# Mononeuritis multiplex: an unexpectedly frequent feature of severe COVID-19

**DOI:** 10.1007/s00415-020-10321-8

**Published:** 2020-11-26

**Authors:** Edward Needham, Virginia Newcombe, Andrew Michell, Rachel Thornton, Andrew Grainger, Fahim Anwar, Elizabeth Warburton, David Menon, Monica Trivedi, Stephen Sawcer

**Affiliations:** 1 Cambridge University Hospital NHS Foundation Trust, Hills Road, Cambridge, CB2 0QQ UK; 2 University Division of Anaesthesia, Department of Medicine, University of Cambridge, Cambridge, CB2 0QQ UK; 3 Department of Clinical Neurosciences, University of Cambridge, Cambridge Biomedical Campus, Cambridge, UK

**Keywords:** COVID-19, Neuropathy, Nerve injury, Mononeuritis multiplex

## Abstract

The prolonged mechanical ventilation that is often required by patients with severe COVID-19 is expected to result in significant intensive care unit-acquired weakness (ICUAW) in many of the survivors. However, in our post-COVID-19 follow-up clinic we have found that, as well as the anticipated global weakness related to loss of muscle mass, a significant proportion of these patients also have disabling focal neurological deficits relating to multiple axonal mononeuropathies. Amongst the 69 patients with severe COVID-19 that have been discharged from the intensive care units in our hospital, we have seen 11 individuals (16%) with such a mononeuritis multiplex. In many instances, the multi-focal nature of the weakness in these patients was initially unrecognised as symptoms were wrongly assumed to relate simply to “critical illness neuromyopathy”. While mononeuropathy is well recognised as an occasional complication of intensive care, our experience suggests that such deficits are surprisingly frequent and often disabling in patients recovering from severe COVID-19.

## Introduction and case series

**Fig. 1:**
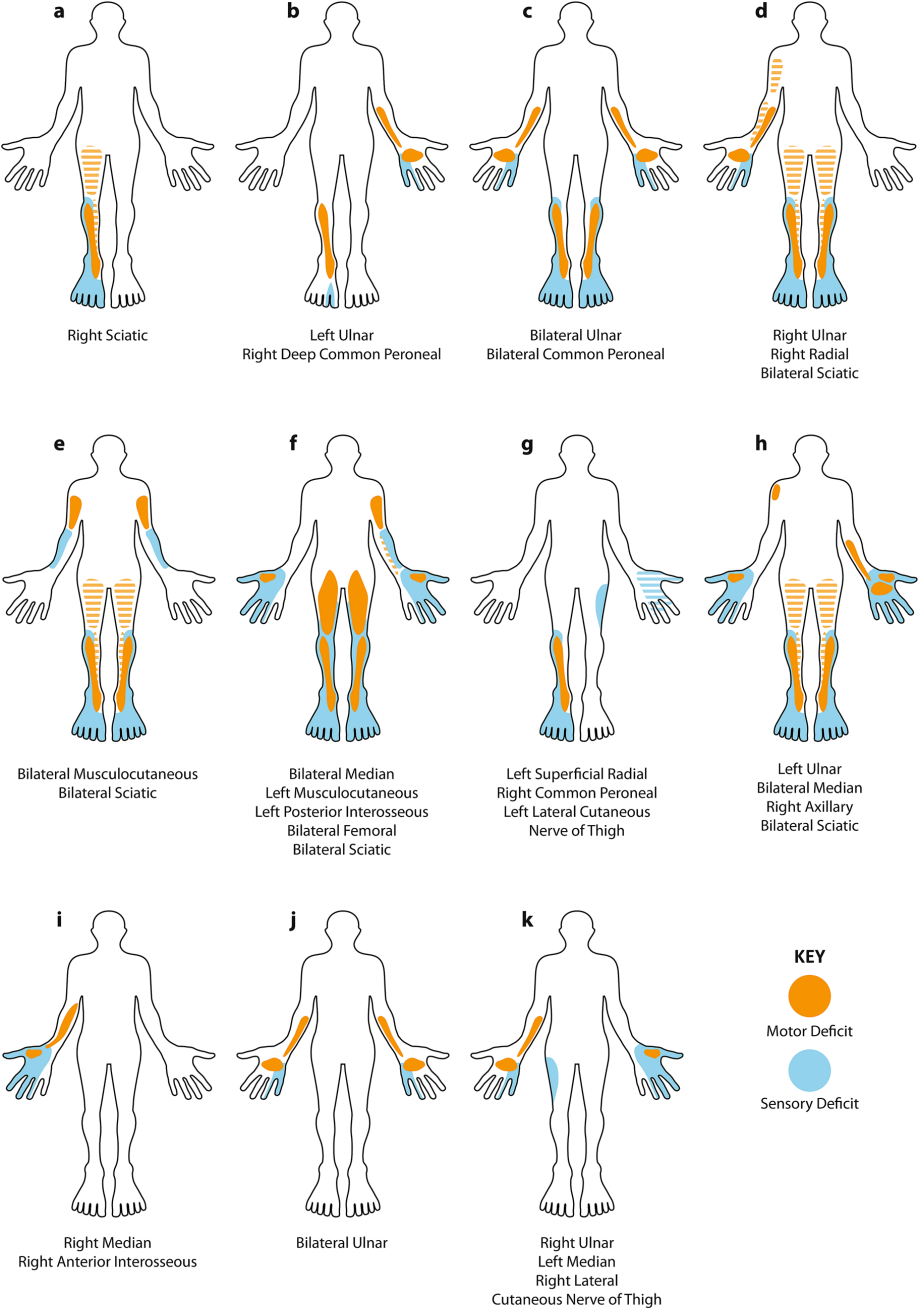
Schematic homuncular illustration of the sensory and motor deficits arising from the multiple mononeuropathies present in 11 patients recovering from severe COVID-19. Hatched shading indicates posterior muscle groups (hamstrings and triceps). In those neuropathies listed as sciatic, there was involvement of both common peroneal and tibial divisions

The respiratory manifestations of the COVID-19 pandemic have strained health-care systems around the world [1]. Thousands of patients have required prolonged periods of mechanical ventilation and many are inevitably emerging with significant “intensive care unit-acquired weakness” (ICUAW) [2, 3]. Amongst the COVID-19 survivors attending our ICU follow-up clinic we have noticed that, in addition to the anticipated symmetrical weakness related to sarcopaenia [4], a significant number of these patients also have marked focal neurological deficits related to superimposed mononeuropathies. Here we present the findings in the first 11 such patients that we have seen (Fig. 1 and Table 1), many of whom are significantly disabled by their neuropathies.

**Table 1:** Demographic and clinical details

Patient	BMI	Proned?	Duration of ventilation	Peak CRP	Peak IL-6	Peak D-dimer
a	26	Yes	38	281	42.6	348
b	26	Yes	31	400	NT	3161
c	22	No	60	350	94.9	11,593
d	30	Yes	26	405	13.2	752
e	53	No	44	268	6.2	382
f	25	Yes	73	314	28.9	14,135
g	21	No	17	523	NT	1059
h	21	No	19	326	380	3199
i	28	No	45	367	28.7	952
j	26	No	16	319	400	3439
k	26	Yes	24	394	3.4	397

NT = not tested

All these patients had severe COVID-19 necessitating mechanical ventilation for an average of 36 days (range 16–73), five required periods of prone positioning, and one required extra-corporeal membrane oxygenation (ECMO). Eight of the 11 were men (representative of the known sex difference for severe disease), and the median age was 58 years (range 50–77). Four of the patients had type 2 diabetes (none on insulin) and most were overweight, the mean baseline BMI being 28 (range 21–53). The neuropathies were noted following withdrawal of sedation, indicating that these nerve injuries developed during the period of ventilation. The clinical deficits have been static or improving when reviewed serially during convalescence, suggesting that the active disease process was limited to the acute phase. In several of the patients, the neuropathies substantially prolonged the need for inpatient rehabilitation and delayed hospital discharge.

All of the patients had evidence of a modest global symmetrical weakness consistent with the sarcopaenia that would be expected following severe critical illness, but none showed electomyographic (EMG) evidence of myopathy. Similarly, only one of the patients showed evidence of concomitant generalised neuropathy (potentially reflecting “critical illness neuropathy” [2]), and this was only mild electrophysiologically. Considering conduction velocity, distal latency, compound muscle action potential (CMAP) duration and F-wave latency, and applying the European Federation of Neurological Societies criteria [5] (originally developed for chronic inflammatory demyelinating polyradiculoneuropathy), we found no evidence of demyelination in any of the affected nerves, with the possible exception of modest conduction block (< 50%) in the right median nerve of one patient. In contrast, all of the patients showed evidence of focal axonal loss with denervation in relevant muscles on EMG and corresponding reductions in both CMAP and sensory nerve action potential (SNAP) amplitudes on nerve conduction testing. Amplitude was reduced to less than 50% of normal and/or less than 50% of the amplitude on the contralateral side in at least one nerve in each patient. Clinically and electrophysiologically, nerve involvement was often patchy, with some nerve fascicles being more severely affected than others. In those patients with partial sciatic neuropathies, it was notable that extensor hallucis longus was invariably the most severely affected muscle, out of proportion to the weakness in tibialis anterior and peroneus longus. Ultrasound scanning of affected nerves was undertaken in three patients and demonstrated diffuse thickening of the affected nerves. The one patient with bilateral femoral neuropathies was found to have bilateral psoas haematomata (a recognised complication of ECMO) on MRI; however, the lower parts of the lumbosacral plexus were uninvolved confirming that these haematomas were not responsible for his sciatica nerve deficits (and obviously did not explain his upper limb neuropathies). Lumbosacral plexus MRI, performed in a second patient with bilateral profound sciatic nerve lesions, was unremarkable: no alternate compressive lesions were identified, and the nerves within the plexus appeared normal. MRI of the brachial plexus in a third patient was also unremarkable; the nerves appeared normal with no evidence of direct injury or avulsion, and no alternative compressive cause identified. As no patient displayed progressive disease, neither nerve biopsy nor lumbar puncture wasundertaken. In the acute phase of their illness, all patients had raised CRP (median 350 [range 268–523]; reference range 0–6), interleukin-6 (median 28.9 pg/ml [range 3.4–400]; reference range 0-2 pg/ml), and d-dimer (median 1059 ng/ml [range 348–14135]; reference range 0-230 ng/ml), but the spectrum of elevation was broad.

At the time of writing, 102 COVID-19-positive patients have been treated in the ICUs in our hospital, 44 of these have been discharged home, 14 have been transferred to other hospitals for further rehabilitation, 11 are still recovering in our hospital and 33 have died. Not all of the discharged patients have yet been seen for follow-up so additional cases of mononeuropathy may still come to light. Whilst mononeuropathies are a well-recognised complication of anaesthesia and intensive care [2], the number of affected patients (at least 16% of those discharged from ICU in our hospital), the number of nerves affected in each patient (an average of just over 3 per patient), and the particular nerves involved (such as the proximal sciatic) are all outside common experience. There have been previous descriptions of bilateral sciatic neuropathies resulting from intensive care, with the patient initially being mistakenly thought to have critical illness neuropathy, but such patients are sufficiently uncommon to warrant individual case reports [6].

## Discussion

This series of cases highlights an important neurological complication occurring frequently in patients with severe COVID-19, which detrimentally affects long-term outcomes and markedly influences their rehabilitation needs. Given that, this complication is evident in a significant proportion of the patients discharged from the intensive care units of a single hospital (16% in our cohort of treated patients) and the rehabilitation burden globally could be substantial. Furthermore, given the high expectation of ICUAW, these focal deficits may go unnoticed.

The underlying aetiology of these neuropathies remains to be established. Mechanical factors related to patient handling and positioning are recognised to occasionally cause focal neuropathy [7], raising the possibility that perhaps patients with severe COVID-19 are very much more susceptible to such nerve injuries. Against this, we saw no evidence for the demyelinating features typically seen in such injuries, and many of our patients had neuropathies at sites that would be highly unusual for compression or traction (including musculocutaneous, proximal median and high sciatic neuropathies). From a clinical and electrophysiological perspective, the neurological deficits we have seen in our patients have much in common with vasculitic neuropathies such as lumbosacral radiculoplexus neuropathy (Bruns–Garland syndrome) and brachial neuritis (Parsonage–Turner syndrome) [8]. However, these syndromes are usually, but not always, proceeded by considerable pain, which was not a common feature in our cohort. However, since our patients developed their deficits while they were sedated and ventilated, this phase of the illness was necessarily obscured. Furthermore, post-mortem studies have confirmed that vasculitis is prominent in a range of tissues in patients dying from COVID-19, although peripheral nerve tissue has not yet been specifically examined [9]. The aetiology of this vasculitis is also uncertain, but might relate to an endotheliopathy [10] resulting from the “cytokine storm” that is so characteristic of these patients [11], or perhaps the microthrombi [12] that are known to frequently occur in COVID-19. The elevations of Interleukin 6 and d-dimer seen in these patients are in keeping with these respective processes, but the broad ranges seen do not clearly support one above the other.

In summary, we have observed a mononeuritis multiplex in a significant fraction of the patients with serve COVID-19 that were admitted to the intensive care units of our hospital. While we recognise this is a small number of individuals in total, and that we have no direct comparative group to demonstrate that this phenomenon is unique to COVID-19, the clinical features are striking and their implications for rehabilitation profound. We strongly urge detailed neurological assessment of patients with post-COVID-19 ICUAW, especially those with asymmetric weakness, as we suspect that many such patient are likely to have focal deficits.

## Data Availability

All available data are included in the paper.
